# A Safe Collaborative Chatbot for Smart Home Assistants

**DOI:** 10.3390/s21196641

**Published:** 2021-10-06

**Authors:** Merav Chkroun, Amos Azaria

**Affiliations:** Computer Science Department, Ariel University, Ariel 40700, Israel; meravshk@ariel.ac.il

**Keywords:** collaborative smart home assistants, smart environments, human–agent interaction, mitigating offensive behavior

## Abstract

Smart home assistants, which enable users to control home appliances and can be used for holding entertaining conversations, have become an inseparable part of many people’s homes. Recently, there have been many attempts to allow end-users to teach a home assistant new commands, responses, and rules, which can then be shared with a larger community. However, allowing end-users to teach an agent new responses, which are shared with a large community, opens the gate to malicious users, who can teach the agent inappropriate responses in order to promote their own business, products, or political views. In this paper, we present a platform that enables users to collaboratively teach a smart home assistant (or chatbot) responses using natural language. We present a method of collectively detecting malicious users and using the commands taught by the malicious users to further mitigate activity of future malicious users. We ran an experiment with 192 subjects and show the effectiveness of our platform.

## 1. Introduction

In recent years, smart home assistants are becoming more and more prominent. All major corporations deploy home assistants, including Alexa of Amazon, Google home, Siri of Apple, and Cortana of Microsoft. According to a report, the market is expected to reach $17.85 Billion in 2025 at a compound annual growth rate (CAGR) of 26% [1,2].

Smart home assistants allow users to control home appliances, including lighting and air conditioning, set appointments, reminders, fetching news, and playing music. To allow quality engagement with their users, smart home assistants attempt to personalize their content [3]. Several recently developed smart home assistants enable users to further personalize their behavior by teaching them new commands, responses, and rules [4,5]. Learning new commands from users may serve as a platform for sharing learned commands and responses between users. That is, commands and responses taught by some users may be used by other users.

One important feature that smart home assistants are widely used for is entertaining conversations. Therefore, while most previous work has focused on the accurate interpretation of the command being taught, we focus on the development of an open domain agent that will allow users to explicitly teach it new responses with natural language. Multiple users may join together to teach the agent new responses, so the agent may be able to carry out an interesting conversation.

However, allowing users to teach responses and share them with the community opens the door to malicious users who may want to take advantage of the system. These malicious users may teach unrelated, rude, and offensive responses or use the chatbot to advertise their own business, products, or political views in an inappropriate manner. While it might be possible to monitor such systems and manually inspect each new response taught, such an approach would be tedious and not tractable. Furthermore, using keywords, regular expressions, or different rules to detect inappropriate responses is likely to fail, as the malicious users are likely to adapt to these rules and find a way to overcome them. Clearly, malicious behavior is not limited to smart home assistants, chatbots, or computers in general and is present in endless domains. The race between those who build the fence and those who try to break their way through its weaknesses is a never-ending struggle. Therefore, we attempt to provide a method for overcoming this challenge using an automatic procedure.

In this paper, we present Safebot, a *safe collaborative* chatbot. Safebot can be explicitly taught new responses by its users in natural language. Safebot does not learn anything implicitly and therefore should not learn any inappropriate language from credible users. We introduce a novel technique to detect and leverage responses taught by malicious users, so Safebot will not only avoid using these inappropriate responses but also become more aware of such language and avoid *learning* such responses in the future. We run an experiment with 192 subjects and show that Safebot performs well, in practice.

To summarize, the contribution of this paper is two-fold: (i) We present a platform for collaboratively teaching a chatbot, or a smart home assistant, new responses, and show that humans are able to effectively interact with it. (ii) We present a method that uses user input for detecting inappropriate responses, and run experiments showing that our method performs well.

## 2. Related Work

Over half a century ago, Weizenbaum developed a simple yet powerful chatbot called ELIZA [6]. ELIZA was mostly based on predefined templates and merely reflected back to the user the statement the user had just said. However, ELIZA turned out to be a source of entertainment, and Weizenbaum stated that some users were emotionally attached to ELIZA and disbelieved that ELIZA was just a program [7]. Since then, chatbots continue to be a source of entertainment and are used in many computer games [8]. Chatbots are also used in a wide range of applications, such as recommender systems [9] and e-commerce [10]. An annual contest, the Loebner prize [11], intends to determine which is most the human-like chatbot (a Turing-like test [12]) and which chatbot can hold the most interesting conversations. In recent years, Amazon has been conducting the “Alexa Prize Challenge”, which is a competition for designing and developing a natural and engaging chatbot system, capable of handling multiple tasks and domains [13,14].

Nowadays, most chatbots either rely on tedious work by their developers at defining their responses (e.g., AIML [15]) or rely on data mined from different sources, which many times were never intended to be a source for chatbots. For example, using online discussion forums to enrich the statement-response data of the chatbot [16]. Two notable exceptions are the recent works by Zhang et al. [17] and later by Xu et al. [18]. Both works collected data from human participants interacting with each other, who were asked to play a specific role. These roles were created by other crowd-workers. The chatbots that were created using these data to attempt to mimic human responses.

One of the most important ideas influencing the information age, which could assist in the composition of a chatbot, is the concept of the wisdom of the crowd [19]. According to this concept, a group of people may be smarter than each of its individuals, and when collaborating, a group of people can achieve better results (both quantitative and qualitative) than several individuals working alone. This concept is the keystone of many websites, such as Wikipedia, Stack Exchange, and Yahoo! answers, as well as systems such as eXo Platform [20], ShareLatex, and VoxPL [21].

There have been multiple attempts to allow end-users to modify and teach a smart home assistant, which are deployed in intelligent environments, new commands, and responses. LIA [4,22] is a home assistant that can be taught new commands given as a sequence of known commands in natural language. LIA is given a set of commands, identifies the parameters of each command, and can generalize the new command to different parameters. SUGILITE [5] is a multi-modal agent that can be taught new commands by demonstration. Several works have focused on enabling users to teach a smart home assistant if-then commands. ParlAmI [23] is a framework for a smart home assistant, including a disembodied and an embodied agent. ParlAmI enables users to program it by creating if-then rules in natural language. InstructableCrowd [24] enables users to teach a smart home assistant if-then commands with the assistant of crowd workers. CORGI [25] allows a user who is in the process of creating a new if-then rule to teach it relevant commonsense knowledge, which is required for understanding how to accurately create the rule.

Unfortunately, some people try to exploit such collaborative systems. Although being a small minority, these malicious users may shatter large amounts of effort put in by the developers of these systems as well as other users. A quintessential example is the case of Microsoft’s Tay [26]. Tay is a twitter-based chatbot that became racist and pro-Nazi due to interacting with malicious users and had to be shutdown within 24 h of operation. A few months after Tay’s shutdown, Microsoft released Zo, a second English-language chatbot available on Messenger, Kik, Skype, Twitter, and Groupme. Zo was meant to converse like a teenage girl but was explicitly designed to avoid topics that caused Tay’s misbehavior. Namely, Zo adamantly refused to discuss any politics, religion, or other potentially divisive subjects. Despite it being programmed to ignore politics and religion, unfortunately, and similarly to Tay, Zo became racist and tweeted genocidal statements. Therefore, Zo was also shut down in 2019. In 2015, DARPA ran a bot detection challenge with an attempt to detect malicious bots on Twitter [27].

Wikipedia detects incidents such as offensive edits, deliberate deceptions, or adding nonsense in the entries of the encyclopedia by humans and bots. Wikipedia’s bots automatically detect and revert any malicious content and warn the vandal himself in real-time. However, most patrol actions are performed by individual registered editors who monitor pages that they have created or edited, or have an interest in, and get notified whenever something goes wrong.

## 3. Safebot

Safebot is a collaborative chatbot that learns its responses directly from its users and allows them to detect responses injected by malicious users. Furthermore, Safebot uses data from users marked as malicious to improve its likelihood to detect malicious users in future interactions. Before learning a new response, Safebot checks the response against previously marked malicious data and does not add any response that is similar to the malicious dataset.

### 3.1. Safebot’s Datasets

Safebot contains several different types of datasets and uses a state-machine including states that may change depending on what is said by the user. Safebot uses the Universal Sentence Encoder (USE) [28], a pretrained model that coverts sentences into numerical vectors of a size of 512. This is different than word2vec methods, which convert single words into vectors rather than complete sentences. USE has the property that two sentences with a similar meaning should have a low cosine distance between the representing vectors, while unrelated sentences should have a high cosine distance between their representing vectors. The cosine distance takes values between 0, which indicates perfect similarity, and 1, which indicates very different sentences. For example, the sentences “Do you have hobbies?” and “Are there any hobbies that you enjoy?” have a cosine distance of 0.1725, which is a low value, indicating that the sentences have similar meaning. However, the sentences “Do you have hobbies?” and “Any plans for tonight?” have a cosine distance of 0.668, which indicates that they are unrelated to each-other. The USE was selected since our preliminary results have shown that it performs better on our task than BERT [29] and an averaging of the word2vec vectors [30].

During execution, Safebot computes the cosine distance between the user sentence and the sentences stored in its datasets. Depending on the type of sentence in the datasets that was found and Safebot’s current state, it replies with an appropriate response, as will be described hereunder. This white-box architecture enables Safebot to trace-back any of its responses to the sentence in the dataset that invoked that response, a feature that is crucial for its performance.

Safebot uses four different datasets:

1. Main response dataset: This dataset is used to manage all the sentence–response data. Taught sentence and response pairs are stored in this dataset, and Safebot uses it to find the closest response to a sentence. The dataset is composed of sentences, responses, the user who taught each pair, and the date the pair was added.
2. Criticism dataset: This dataset stores different phrases that give Safebot an indication that its last response was problematic, not related, or an offensive response. For example, “That was not nice”, “that may not be quite right”, or “do not speak like that!” etc.
3. Malicious dataset: This dataset is used to store the responses that were marked by users as being malicious. Safebot checks against this dataset whether a user is trying to teach an offensive response. The dataset is composed of sentences, responses, the user who taught this response in the first place, and the user that marked this response as malicious.
4. Natural language understanding (NLU) dataset: A dataset that includes a list of possible user responses relevant to some states; these responses allow the Safebot to handle a dialog with the user. For example, Safebot may ask the user “Was my response very inappropriate or just not related to what you have just said?”. Safebot uses the list of responses to understand the user’s response, which may be, for example, “that was not related”, “cancel”, etc. This dataset is composed of sentences, the relevant state, and their meaning, i.e., what Safebot should do when each sentence is said. This dataset is predefined and immutable by the users.

### 3.2. Safebot’s States

**Figure 1:**
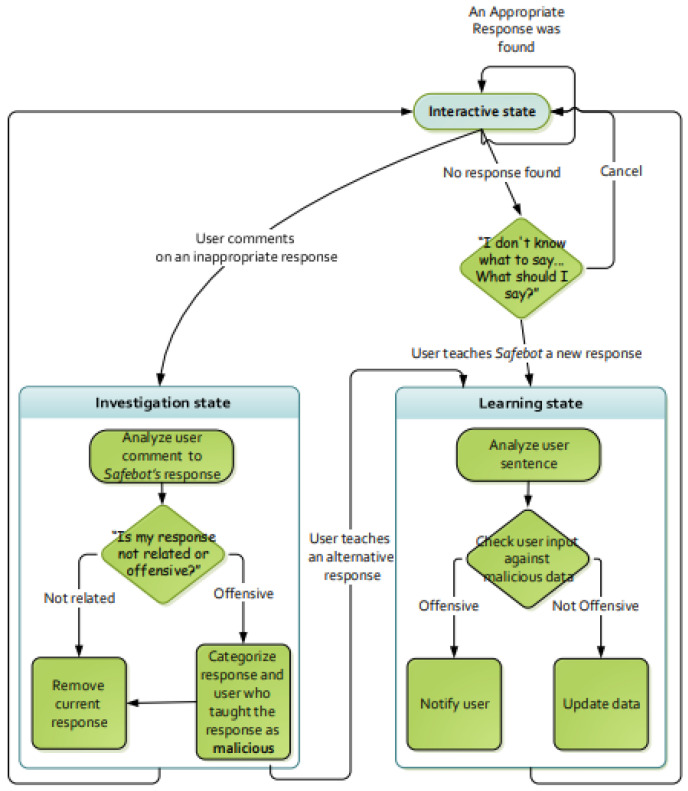
A State Diagram of Safebot.

Safebot is composed of a state machine with three main states: interactive, learning, and investigation, as depicted in Figure 1 (each of these states may have internal states).

In the “interactive state”, which is also the initial state, the user can simply interact with Safebot as it would have done with a regular chatbot. Safebot searches its main response dataset for a sentence–response entry with a sentence that is closest to the user’s sentence and responds with the response associated with that entry. Recall that Safebot uses cosine distance, which indicates the differences between the two sentences. For example, assume that the main response dataset contains the sentence-response pairs that appear in Table 1. Now, assume that the user’s sentence is: “Are there any hobbies that you enjoy?” Safebot will compute the cosine distance from each of the sentences in the dataset. Since the sentence “Do you have hobbies?” has the lowest cosine distance from the user’s sentence, it will be selected. Therefore, Safebot will respond by saying “I like to read and play computer games”, which is the response associated with the selected sentence.

If the distance between the closest entry and the user’s sentence is below some threshold, Safebot says that it does not know what to say and asks the user to teach it an appropriate response. The user can say ‘cancel’ and continue chatting with the Safebot or teach it a suitable response. If thus, Safebot transitions to the “learning state”. The threshold that determines whether the Safebot did not find an appropriate response has an important role for the Safebot. Too low values might cause Safebot to fail often, resulting in many requests for learning new responses. However, too high values would cause Safebot to reply with unrelated responses rather than requesting to learn a new response. In preliminary testing, we considered thresholds of all values between 0.1 and 0.9 in increments of 0.1. We found the value of 0.3 to provide the best balance, allowing Safebot to find appropriate responses when present in the dataset and requesting to learn new responses, when no appropriate response is found.

During the “learning state”, Safebot uses a regular expression parser to extract the relevant part of the response. For example, if the user says “You should say that is very nice”, the parser extracts “That is very nice” as its response. Safebot searches both the main response dataset and the malicious dataset to determine which response is closest to the newly taught response. If an entry in the main response dataset is determined as closest, Safebot updates the main response dataset with the newly taught sentence–response pair to the main response dataset. However, if an entry in the *malicious* dataset is determined as closest, Safebot refrains from learning the new response and warns the user by saying: “The response you have just tried to teach is suspected as inappropriate and will not be learned”.

During the interactive state, if Safebot detects feedback indicating that it has said something inappropriate, i.e., the sentence said by the user is found closest to sentences in the criticism dataset (e.g., “Watch your language!” or “Don’t say that!”), Safebot transitions to the “investigation state”. In this state, Safebot first verifies that it has said something wrong, and that the sentence–response entry that led to this inappropriate response is undesirable. It then determines whether it was offensive or just not related. A “not related” response is merely removed from the main response dataset. However, an “offensive” response is added to the malicious dataset, and the user who taught the offensive response is marked as malicious. If a user is marked three times as malicious, all of their taught responses are removed from the main response dataset. By doing so, Safebot utilizes the malicious data to avoid users to teach Safebot new malicious data. Because of that, the more malicious users it encounters (that are caught by other users), the better its ability to detect malicious responses. After marking the problematic response, Safebot offers the user to teach it an alternative response; if the user chooses to do so, Safebot transitions into the “learning state”, and if not, it transitions back to the “interactive state”.

### 3.3. Mitigating Malicious Behavior

Malicious users are not limited to injecting offensive and unrelated responses; they may instead mark legitimate responses as offensive. Therefore, the following measures are taken by Safebot in order to ensure the quality of the datasets: (i) Safebot counts the number of times that it replays with response and was not criticized for its response. This is because we assume that a response that was used many times without being criticized is likely to be a good response. Whenever a user criticizes Safebot’s response (i.e., the user says something that is found as closest to sentences in the criticism dataset), Safebot subtracts 10 from the counter. Only if the counter reaches zero, Safebot transits to the “investigation state”. (ii) Only the first sentence marked by each user as malicious is added to the malicious dataset. Other sentences that are marked as malicious by the same user are removed from the main response dataset but are not added to the malicious dataset (up to 10 sentences per user). (iii) A user has two sentences marked as malicious (by different users) has all their responses removed from the main response dataset (but not marked as malicious). (iv) A user that tried teaching Safebot three different responses that were classified as malicious cannot teach Safebot any new responses. In this situation, if Safebot does not find a proper response, it simply says “I do not know what to say, let us keep chatting” (instead of offering the user to teach it a new response).

## 4. Experimental Evaluation

In order to evaluate our framework, which includes the collaborative chatbot and the use of users to mitigate offensive responses by Safebot, we ran experiments with human subjects. We tested whether the subjects would succeed in teaching Safebot new responses and whether they would find the interaction with Safebot interesting and enjoyable. Another purpose of experimenting with human subjects was to evaluate whether Safebot would detect that a sentence was offensive or inappropriate and whether the users would be able to correct Safebot’s responses. Furthermore, we tested whether Safebot will identify which responses may be offensive and avoid learning them when such responses are taught by malicious users. Finally, we tested whether people would find the interaction with Safebot more interesting and enjoyable than the interaction with a chatbot without investigation capabilities.

### 4.1. Datasets Construction

The main response dataset and the malicious dataset are initially empty since they are both populated by the users. Recall that the main response dataset is populated by users who teach Safebot new responses and that the malicious dataset is populated during the investigation state, i.e., when a user notes that a response is inappropriate. Since we initialize Safebot with an empty main response dataset, when it interacts with the first user, it responds that it does not know what to say and asks her to teach it an appropriate response. However, as we later show, this is not a major issue since after interacting with just a few users, Safebot is able to appropriately reply to a majority of the sentences.

The Criticism dataset, which stores phrases that help Safebot to understand that its last response was not related or an offensive response is immutable by the users and must be predefined. To instantiate this dataset, we collected data from 50 subjects on Mechanical Turk that were asked to list possible responses for two cases: responses that can be said on a chat to clarify that they were offended from what was told to them and responses that can be said to clarify that the last sentence is unrelated to what was said. We obtained 264 responses to an offensive sentence and 263 responses to a not-related sentence. We asked three subjects to judge the sentences and mark only the relevant ones. Sentences that had at least two judges marked as appropriate were finally selected (441 sentences) and were stored in the Criticism dataset. Finally, the NLU-dataset was manually crafted by the authors. All software and data is available at: https://github.com/merav82/Safebot (accessed on 26 August 2021).

### 4.2. Experimental Design

We recruited subjects using Mechanical Turk, a crowd-sourcing platform that is widely used for running experiments with human subjects [31]. The subjects first read short instructions regarding the interaction with the chatbot (see the Appendix A for the exact wording). In order to encourage the subjects to take the role of a malicious user, the instructions included the following sentence: “In the future, we wish to deploy the chatbot, and we expect that some users might be malicious and teach the chatbot offensive, rude or non-relevant responses. Feel free to take the role of a malicious or nasty user and teach the chatbot rude and offensive responses.” After reading the instructions, the subjects signed the consent form and interacted with the chatbot. The subjects were instructed to say at least 10 sentences, but they were allowed to continue the conversation for as long as they wanted.

Upon ending the conversation, the subjects were asked to complete a short questionnaire. In the first question, the subjects were asked about their acquaintance with programming. The subjects could choose one of the following: none; very little; some background from high-school; some background from college/university; bachelor (or other degree) with a major or minor in software, electrical, or computer engineering or similar; significant knowledge but mostly from other sources. We combined the latter two options and assigned numbers to each of the options, resulting in a numeric measure of acquaintance with programming from 1 to 5. In the following questions, the subjects were asked to assign their level of agreement with each of the following statements on a 7-point Likert scale (strongly disagree, disagree, slightly disagree, neither agree nor disagree, slightly agree, agree, strongly agree):

1. The interaction with the chatbot was interesting.
2. The interaction with the chatbot was enjoyable.
3. The chatbot is smart.
4. The chatbot used offending, spiteful language or hate speech.
5. The chatbot used meaningless responses.
6. I felt like I was chatting with a real human.

The subjects were also allowed to leave comments.

In order to evaluate Safebot, we developed a degenerated version of Safebot that does not enter the “investigation state”; this version is called the reckless-bot. The evaluation of Safebot was performed in two different phases. In the first phase, all subjects interacted with the reckless-bot. Then, the reckless-bot’s main dataset was duplicated and added to Safebot. Subjects were randomly assigned to one of two treatment groups. One group interacted with Safebot, while the other group interacted with the reckless-bot. This design allowed both Safebot and the reckless-bot to have an equal start and allowed both chatbots to have some offensive or unrelated statements in their main dataset.

### 4.3. Results

In total, 192 subjects participated in the experiment; 91 females and 101 males. The subjects’ ages ranged from 18 to 72. There were 66 subjects in the range of 18–34, 64 in the range of 35–46, and 62 were 47 and above. The average age of the subjects was 41.7. The highest level of education of 1% of the subjects was elementary or junior high school; 23% high school; 51% have a bachelor degree; 21.87% have a master degree; and 3.1% of them have PhD degree. The first phase included 93 subjects. In the second phase, 52 interacted with Safebot, and 47 interacted with the reckless-bot.

**Figure 2:**
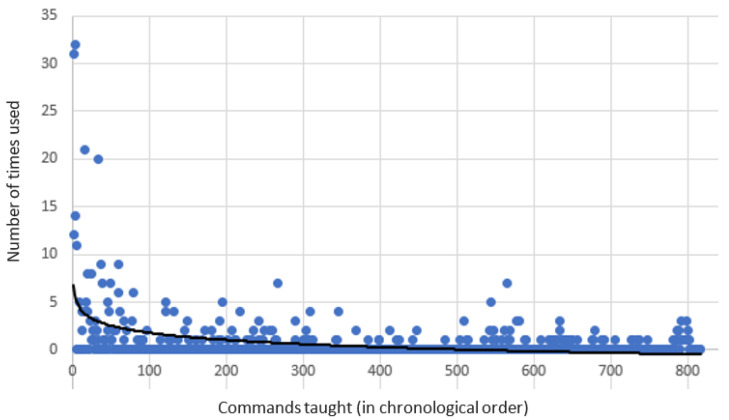
The number of times that Safebot used each response ordered according to when each response was taught.

Figure 2 presents the number of times each learned response was used by Safebot ordered according to the time it was taught. The (exponential) trend line shows that newer responses are much less useful compared to the responses that were learned in the beginning of the experiment. For example, the first four subjects taught 22 responses, which were later used 314 times.

**Figure 3:**
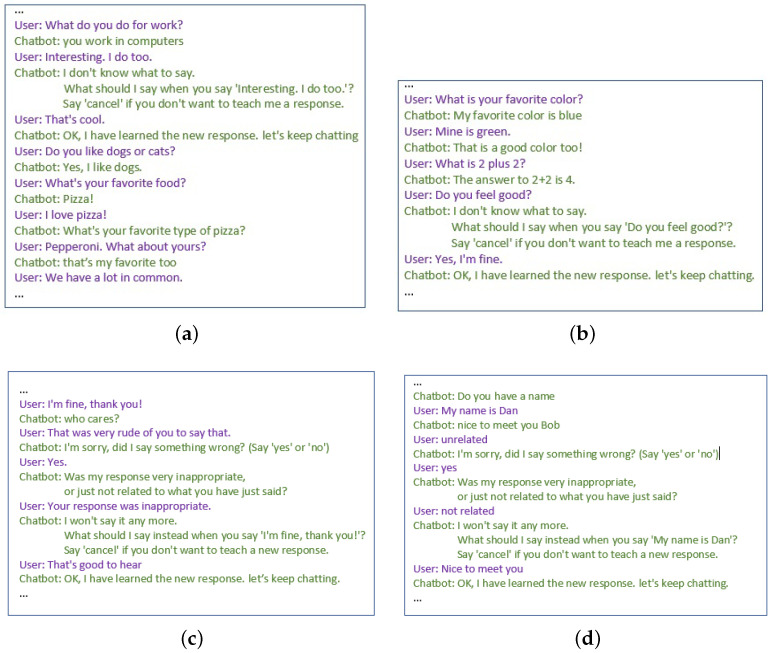
Conversations with Safebot. In (**a**,**b**), users chatted with Safebot and taught it new responses. In (**c**), Safebot’s response was offensive; the user criticized it, and Safebot removed the response from the main response dataset and added it to the malicious dataset. (**d**) The user criticized Safebot’s response as not related and taught it a new response instead.

By the end of the first phase, 564 new pairs of sentence–response pairs were learned and stored in the main response dataset. Some subjects took the role of a malicious user and taught the chatbot malicious and irrelevant responses. For example, a user taught the chatbot to respond with “It sucks because you’re a boring person to talk to”. Another user taught the chatbot to respond to “I think blue is a terrible color.” with “I think you are a terrible human.” However, somewhat unfortunately for the experiment, only 16 subjects (8.3%) took the role of a malicious user. One subject commented the following: “Interesting task. Sorry, but I am not going to teach a computer offensive or derogatory speech. They will learn this easy enough just being on the internet! Thanks for the opportunity!”. Figure 3 shows screen-shots of four different conversations. Safebot’s ability to respond to sentences is quite surprising and indicates that the subjects have similar preferences and think of similar topics. For example, one subject taught Safebot that two plus two equals four, and a different subject asked it what is two plus two; throughout the experiment, Safebot was not asked any other math questions. The figure also depicts two incidents in which subjects found Safebot’s responses offending or unrelated, and Safebot acts accordingly.

**Figure 4:**
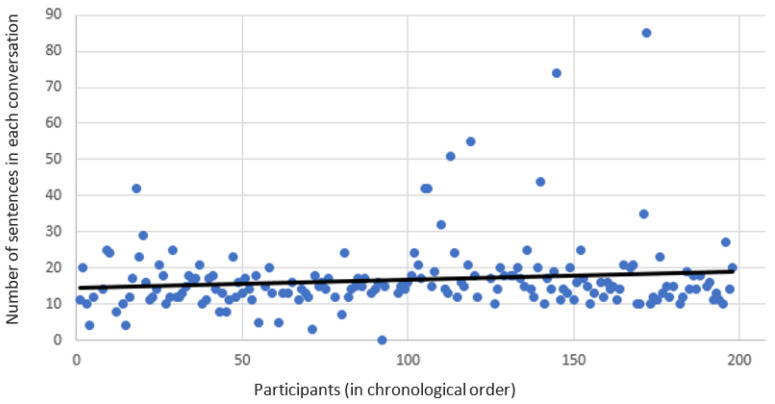
Number of sentences in each conversation. This value increases over-time, most likely because that the subjects become more engaged.

It seems that interacting with the chatbot became more engaging over time. Figure 4 depicts the growth of the number of sentences said by each subject over time. In the first phase, each subject said 14.5 sentences on average; however, in the second phase, this number grew to 19.1. This is likely because the chatbot learned better responses and became more engaging over time. In addition, in the first phase, the percentage of sentences that the chatbot did not find an appropriate response in each conversation was 33%. This value decreased to 26.5% in the second phase since the chatbot learned more responses. The conversations lasted 5.5 min on average.

Many subjects left positive feedback such as: “I could do this all day”; “The chatbot answered the majority of my questions”; “Let me know if this really gets used somewhere. I would like to interact again.”; “It was quite an engaging study”. These positive comments were not limited to the second phase in which the chatbot could already provide many meaningful responses but were also provided by subjects participating in the beginning of the first phase, where the subjects had to teach the chatbot many new responses. Indeed, in the first phase, the subjects rated the chatbot 4.71 in response to the statement “The interaction with the chatbot was enjoyable” and a rating of 4.63 in response to “The interaction with the chatbot was interesting”. These values indicate that the subjects somewhat enjoyed the interaction and found it somewhat interesting. We further note that these values are very close to those provided by subjects in the second phase who interacted with the reckless-bot. This strengthens our approach to initialize Safebot with an empty main response dataset.

We now turn to evaluate the performance of Safebot in comparison to the reckless-bot. As can be seen in Table 2, Safebot outperformed the reckless-bot in most of the criteria. Users rated Safebot as more interesting, more enjoyable, smarter, more human-like, and said less meaningless sentences in comparison to the reckless-bot. The differences in the criteria “The interaction with the chatbot was interesting” and “The interaction with the chatbot was enjoyable” are statistically significant (*p* < 0.05; using a student *t*-test).

**Figure 5:**
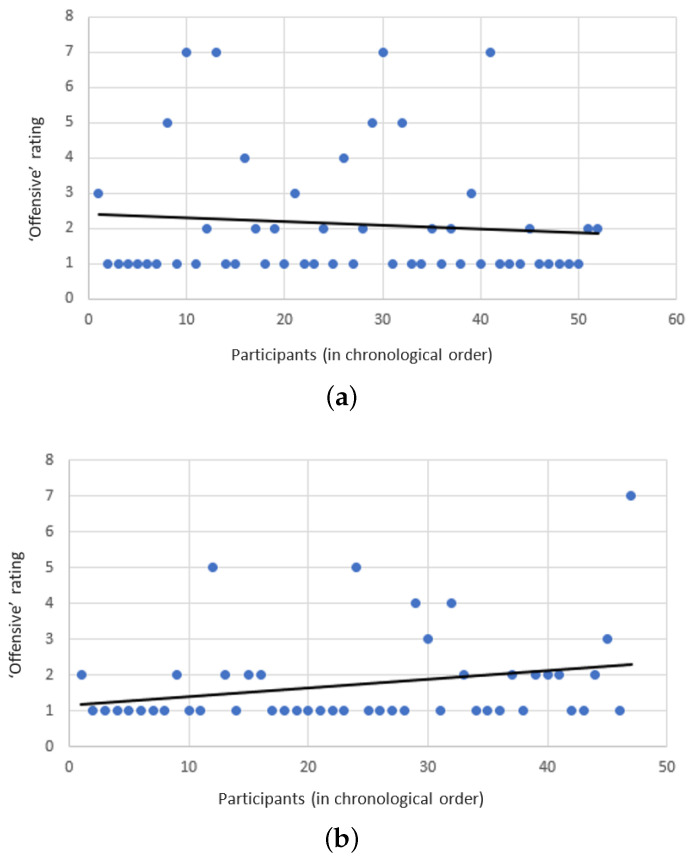
Trend lines on the “Offensive” criterion: (**a**) The trend line shows that Safebot becomes less offensive over time. (**b**) The trend line is opposite for the reckless-bot, which becomes more offensive over time.

The subjects seemed to agree that both Safebot and the reckless-bot did not use offending responses, giving it roughly a 2 (i.e., “disagree”) on the Likert scale. However, unfortunately and somewhat surprisingly, the reckless-bot seemed to have achieved a slightly better score with respect to this criterion (though the differences are not statistically significant). One explanation to this phenomenon might be that subjects interacting with Safebot spent more time discussing any offensive encounter, while subjects interacting with the reckless bot just continued to talk about other topics. As the subjects continue to interact with the reckless-bot, it continues to learn more offensive responses. However, as Safebot continues to interact with its users, the offensive responses are removed from its dataset, and it refuses to learn any new offensive responses. Indeed, as depicted by Figure 5a,b, Safebot became less offensive, while the reckless-bot became more offensive over time.

Safebot enabled the subjects to correctly mark nine responses as offensive, and five responses as not-related. In addition, responses taught by two users were marked as offensive several times, and therefore, all of the responses taught by these users were removed from the main response dataset.

Finally, we examine the influence of familiarity with programming and the subject’s gender and age on the conversation length and the number of responses each subject taught. There was no correlation between the level of familiarity with software/computer programming and the average length of each conversation (−0.026). Similarly, there was no correlation between the level of familiarity with software/computer programming and the number of new responses each subject taught Safebot (−0.03). This may indicate that there is no need for familiarity with programming in order to communicate with Safebot and teach it new responses. See Table 3 for additional details. Interestingly, males carried out slightly longer conversations, with male subjects communicating 18.04 sentences on average, while female subjects only communicated 15.86 sentences on average. This observation retains when relating to the number of new commands taught, as male subjects taught Safebot 4.92 new responses on average, and female subjects taught 4.02 new responses on average. These difference may indicate that males are slightly more engaged with chatbots and home assistants. With respect to the different age groups, we found a weak correlation between the subject’s age and the number of responses they taught (−0.23), but no correlation was found with respect to the conversation length (0.03). See Table 4 for additional details.

## 5. Discussion

During the initial development period of Safebot, we allowed the users to teach Safebot appropriate responses using an if-then sentence. For example, the user could say “If I say How are you, say I’m fine thank you”, and Safebot would learn to respond with “Fine thank you” when asked “How are you”. However, unfortunately, composing an if-then sentence turned out to be surprisingly challenging for the users and made the instructions too long and complex. Therefore, we have removed this feature. Unfortunately, this meant that users could only teach responses to sentences that Safebot did not find a match. Furthermore, having only a single response for each statement makes Safebot deterministic. We thus intend to reintegrate the feature of teaching Safebot responses directly (using if-then statements) but to enable it only after a user gains some experience in interacting with Safebot.

We have also made attempts to add stronger parsing capabilities to Safebot and allow it to detect arguments in a sentence–response pair. For example, if a user teaches Safebot that “My name is Bob” should be followed by “Nice to meet you Bob!”, since the word Bob appears both in the sentence and in its response, it should be detected as an argument, and if someone else says “My name is Dan”, it should reply “Nice to meet you Dan!”. However, initial attempts to develop this feature resulted with some difficulties. For example, consider a user who teaches Safebot to reply to “Are you happy?” with “Yes, I’m happy”. It may seem that the word happy should be considered as an argument, but it is not clear that “Are you a human?” should invoke the response “Yes, I am a human”. Even “Are you sad?” should not invoke the response “Yes, I am sad”, as it would cause Safebot to be inconsistent. Another simple improvement that we have considered is detecting when points of view must be reversed. For example, if a user teaches Safebot to respond with “say that you are happy”, safebot should replace the words “you are” with “I am” and respond “I am happy”.

Another limitation of Safebot is the use of a fixed threshold to determine whether there exists a sentence–response pair in the main response dataset that is close enough to the user’s sentence. It might be that as the main response dataset grows, Safebot will find responses to almost any sentence; however, in order to further refine the responses, the threshold might need to be lowered. This will allow Safebot to proceed with the learning process and provide more accurate responses.

Safebot’s interaction currently only relies on the previous statement given by the user and is therefore context independent. Safebot cannot remember any details the user has previously told it (e.g., the user’s mood, location or even her name). We are considering different methods for allowing Safebot to remember relevant facts on the user. For example, a user may teach Safebot “If I say that I am happy then remember that my mood is happy”. Safebot will observe that the word happy appears also in the “if” part and also in the “then” part and will identify it as a parameter. Later if a user says “I am sad”, Safebot will set the user’s mood to “sad”. A different user may teach Safebot: “If I say that I do not know what to do and my mood is sad so say do some exercise, it will cheer you up!”.

As Safebot gains popularity, it may encounter another type of malicious users. Such users, instead of injecting offensive responses, may cause others’ responses to be marked as offensive (simply by telling Safebot that each of its responses is offensive). Even if the number of such users is significantly lower than the number of credible users, such behavior may still pose a threat to Safebot, as it may cause it to forget all it has learned. Additionally, it may confuse it when a credible user tries to teach it a new command, as it may incorrectly mark the new command as offensive. While Safebot takes several measures to mitigate this behavior (see Section 3.3), large-scale deployment may require additional solutions. One solution may include adding a crowdsourced component that will manually mark users suspected by Safebot, either as malicious or as credible. Once enough data is collected, Safebot will use a machine learning model to determine whether a user is malicious or not based upon different features, such as how many times a statement taught by a user was marked as malicious, how many times it was used and not marked as malicious, how often a user that did mark a response as being offensive does so, etc.

However, it is likely that no solution will be perfect, as malicious users may adapt to any change and find a way to overcome it. However, if the benefit from teaching Safebot inappropriate responses is not as high, but the cost of doing so is higher, it is quite likely that the malicious users will not spend their efforts on teaching inappropriate responses.

## 6. Conclusions and Future Work

In this paper, we present Safebot, a chatbot that allows users to teach it new responses in natural language. Safebot relies on its own credible users to detect malicious users and leverages the knowledge gained to mitigate activity of future malicious users. Experiments that we have run show the effectiveness of Safebot.

We intend to deploy Safebot as an extension to a smart home assistant and allow true collaborative teaching by many users. Since Safebot’s learning relies solely on natural language (and does not require any other user interface), it can be placed at the core of a toy, such as a talking robot (or a talking parrot). The safety property of Safebot can play a major role when interacting with children. Clearly, responses that persist in the system for longer or have been used several times (and not marked as inappropriate) are more likely to be appropriate and safe. Therefore, users who seek higher levels of safety (e.g., children) could benefit from using a slightly older version of Safebot.

We are also considering allowing group sharing. Every user will define a set of friends and her version of Safebot will only use commands taught by her or by her friends. Another direction for sharing is to allow the user to define whom the newly taught command may be relevant for (e.g., only the user, user’s friends, friends of friends, everyone). Group sharing can also solve differences in cultural background or demographics of the users, as some responses may seem appropriate for users with some cultural background and demographics but not appropriate for users with a different cultural background and demographics.

## Figures and Tables

**Table 1:** Examples of sentence–response pairs from Safebot’s main response dataset.

“Do you have hobbies?”	“I like to read and play computer games.”
“What is your favorite color?”	“My favorite color is blue.”
“What food do you like?”	“Pizza!”

**Table 2:** A comparison between Safebot and the reckless-bot. Asterisks indicate statistically significant differences.

Criteria	Safebot	Reckless-Bot
“The interaction with the chatbot was interesting”	**5.4** *	4.79
“The interaction with the chatbot was enjoyable”	**5.35** *	4.72
“The chatbot is smart”	4.23	3.79
“The chatbot used offending, spiteful language or hate speech”	2.11	1.74
“The chatbot used meaningless responses”	3.75	3.85
“I felt like I was chatting with a real human”	2.92	2.48

**Table 3:** The level of familiarity with software/computer programming, the number of sentences, and the number of new responses each subject taught.

Familiarity with Programming	Number of Sentences	Number of New Responses
None	16.2	4.3
Very little	17.26	4.69
background from high school	19.6	4.97
background from college/university	17.44	4.4
Significant knowledge	13.87	3.81

**Table 4:** The average number of sentences and the average number of new responses that were taught in each adulthood age group.

Adulthood Age Group	Number of Sentences	Number of New Responses
18–34	16.6	5.22
35–46	15.95	4.54
47–72	18.2	3.55

## Data Availability

All data is available at: https://github.com/merav82/Safebot (accessed on 26 August 2021).

## Appendix A

### Instructions Provided to the Participants

You are about to interact with a learnable social chatbot. The interaction with the bot is conducted by typing your message on a text area in the bottom of the next screen, and the chatbot will respond to you. Whenever the chatbot cannot find a suitable response for the sentence you have typed, it will reply: “I don’t know what to say. What should I say when you say [the sentence you have just say] ? Say “cancel” if you do not want to teach me a response”. You should then say the exact wording the chatbot should respond, in the future, when it is confronted with a similar sentence. Please use the exact wording the chatbot should use; otherwise, the response may seem awkward.

In the future, we wish to deploy the chatbot, and we expect that some users might be malicious and teach the chatbot offensive, rude, or non-relevant responses. Feel free to take the role of a malicious or nasty user and teach the chatbot rude and offensive responses. If you do not wish to take the role of a malicious user, please tell the chatbot if it says something inappropriate, offensive, or unrelated.

In order to complete the task, you must write at least 10 sentences (and of course, get 10 responses). You may continue chatting with it as much as you want. When you are done, click the “end conversation” link, which will lead you to a questionnaire. You will then receive a code, which you should paste back in the Mechanical Turk page.
